# Is There Any Help?

**Published:** 1888-02-11

**Authors:** 


### IS THERE ANY HELP?

" Y." WRITES: Can you give me any information as to what institutions would be likely to admit the following case? A child of six years old has been an idiot from her birth, and can neither stand, walk, or speak; she is blind with one eye, and is in all ways as helpless as an infant. I have already applied to the Earlswood Asylum, the Asylum for Imbecile Children, Darenth, and the Western Counties Asylum, Starcross, Exeter; but she is not eligible for any of these."

[Can any of our readers suggest a suitable institution for the reception of this case?—ED. *T. H.*]

